# Histidine Tag-Specific PEGylation Improves the Circulating Half-Life of TIMP2

**DOI:** 10.1021/acsabm.4c01385

**Published:** 2025-02-21

**Authors:** Jack Toor, Wiktoria R. Grabowska, Adam L. Johnson, Jane Jones, William G. Stetler-Stevenson, Hanieh Khalili, David Peeney

**Affiliations:** † Laboratory of Pathology, Center for Cancer Research, National Cancer Institute, Bethesda, Maryland 20892, United States; ‡ School of Medicine and Biosciences, University of West London, London W5 5RF, U.K.; § Protein Expression Laboratory, FNLCR, NIH, Frederick, Maryland 21702, United States; ∥ School of Pharmacy, University College London, London WC1N 1AX, U.K.

**Keywords:** TIMP2, PEGylation, pharmacokinetics, biologics, therapeutics

## Abstract

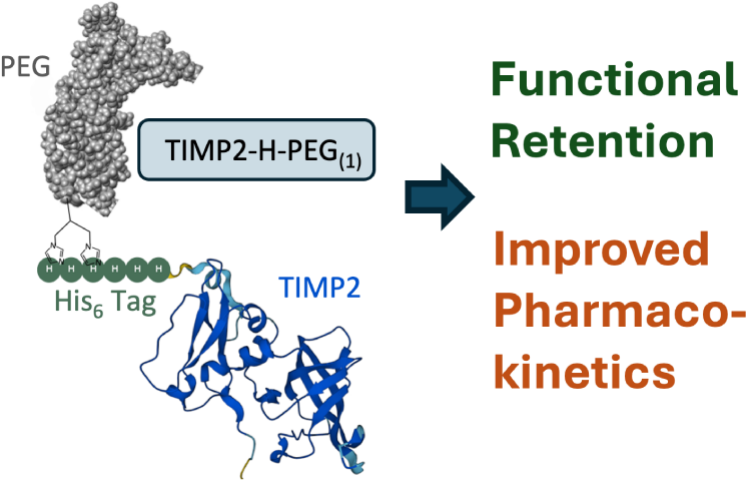

An overarching limitation of therapeutic biologics is the limited half-life these proteins often exhibit once in circulation. PEGylation, the chemical conjugation of proteins to poly(ethylene glycol) (PEG), is a common strategy to improve protein pharmacokinetics (PK) by enhancing stability, reducing immunogenicity, and decreasing renal clearance. Tissue Inhibitor of Metalloproteinases 2 (TIMP2) is a 22 kDa matrisome protein that exhibits therapeutic potential across a range of human disease models yet possesses a short serum half-life. To advance the therapeutic development of recombinant His-tagged TIMP2 (TIMP2), we utilized primary amine conjugation (1 kDa) and site-specific histidine conjugation (10 kDa) to improve its circulating half-life. Primary amine conjugation of PEG molecules to TIMP2 (TIMP2-a-PEG(_*n*_)) is efficient, yet it produces multiple positional isomers that are difficult to purify. Furthermore, high levels of conjugation can affect the MMP-inhibitory activity of TIMP2. Despite this, TIMP2-a-PEG(_*n*_) displays a significant improvement (11.5-fold) in serum half-life versus unconjugated TIMP2. In contrast, site-specific histidine conjugation targets the histidine tag, enabling the purification of mono-PEGylated (TIMP2-H-PEG_(1)_) and di-PEGylated (TIMP2-H-PEG_(2)_) forms. Our findings demonstrate that TIMP2-H-PEG_(1)_ exhibits improved PK with enhanced stability and a 6.2-fold increase in circulating half-life while maintaining MMP-inhibitory activity. These results suggest that site-specific PEGylation at a C-terminal His_6_ tag is a promising approach for further preclinical development of TIMP2 as a therapeutic biologic.

## Introduction

Tissue Inhibitor of Metalloproteinases 2 (TIMP2) is a widely expressed 22 kDa matrisome protein that exhibits promise as a therapeutic in multiple human disease models, traversing cancer, neurological, and vascular disorders.^1^ Classically, it functions as a metalloproteinase inhibitor that binds to and inhibits the proteolytic activity of all members of the matrix metalloproteinase (MMP) family, in addition to specific members of the A disintegrin and metalloproteinase (ADAM) and ADAM with thrombospondin motifs (ADAM-TS) families.^2^ Beyond its role as a metalloproteinase inhibitor, TIMP2 has various additional functions that coalesce around antimitogenic and immunomodulatory activity in tissues.^2−6^ These findings helped establish the idea that the features of TIMP2 biology may harbor therapeutic benefits in a host of human diseases.^1^ Indeed, we have previously reported that daily administration of TIMP2 is beneficial in murine models of breast and lung cancer,^4,7^ and others have described the utility of purified TIMP2 as a treatment in various other disease systems.^8−11^ Like most biologics that are not typically serum proteins, the therapeutic utility of TIMP2 is restricted by its short circulating half-life. Rapid clearance from the circulation makes it difficult to determine dose-response relationships and requires much larger doses of native protein.

Various methods can be employed to improve the pharmacokinetics (PK) of therapeutic proteins, including conjugation with poly(ethylene glycol) (PEG) polymers, direct fusion with serum proteins, and polypeptide extension (PE). PE can have unique modes of action, mediated through affinity to serum proteins^12^ or through a long, disordered amino acid tail (Pro, Ala, Ser amino acids, PASylation) that improves PK via expansion of the molecule’s hydrodynamic volume.^13^ Many of these methods have been applied to full-length TIMP or TIMP domain proteins, such as fusion with serum proteins^8,14^ or PEGylation at primary amines and other specific sites.^15,16^ Furthermore, a method similar to PASylation (PATylation) was recently utilized with the *N*-terminal domain of TIMP2 to deliver a 3.5-fold increase in serum half-life.^17^ Each of these methods has distinct benefits and drawbacks, reviewed extensively elsewhere.^18−20^ PEGylation is the most common and diverse method that can be utilized for improving PK, achieved by an improved circulating half-life, increased stability, and/or reduced immunogenicity (reviewed elsewhere).^21−23^ These benefits are derived through an increase in the hydrodynamic size of the protein, a feature that is linked to the size of the PEG when in linear form.^24^ The simplest means for protein PEGylation is through primary amine conjugation using succinimidyl (NHS) ester functional groups conjugated to PEG.^21^ Maleimide–thiol (cysteine) conjugation is another popular method of PEGylation in more restricted locations due to the rarity of cysteine residues in proteins (approximately 1%).^25^ TIMP proteins contain 12 cysteines contributing to 6 disulfide bridges that are crucial to the TIMP structure, indicating that a free cysteine would need to be introduced to the TIMP sequence to utilize this method. This was attempted by Batra et al., who introduced a free cysteine at the C-terminus of TIMP1 for PEGylation. However, due to cysteine oxidation and the incompatibility of reducing agents with TIMP1 structure–function retention, this method proved unsuccessful.^15^ While not yet widely adopted, histidine PEG conjugation offers a valuable strategy for site-specific PEGylation, which relies on the use of PEG-*bis*-sulfones through *bis*-alkylation chemistry.^26,27^ This versatile chemistry also allows for the rebridging of the thiols from disulfide bonds present in the protein after an initial reduction step.^28^ Notably, histidine-PEGylated biologics demonstrate strong retention of their original bioactivity,^27^ and the simple His-*x*-His target sequence provides researchers with a highly flexible and tunable system for modification.

We demonstrate that PEGylation is an effective means of improving the PK of therapeutic TIMP2. We have utilized two PEGylation methods: nonspecific conjugation through amine groups and site-selective conjugation at histidine target sequences. Amine reactive conjugation is highly efficient and greatly improves the circulating half-life of TIMP2. However, this method is random, producing excessive numbers of positional isomers that are problematic for quantification and purification. Further, we show that TIMP2 is an excellent candidate for site-specific PEGylation using PEG-*bis*-sulfone, from which mono- and di-PEGylated populations can be purified. Mono-PEGylated TIMP2 (TIMP2-H-PEG_(1)_) exhibits improved PK and functional retention, representing a key milestone in the continued development of TIMP2-based therapeutics.

## Results

### 1 kDa PEG Conjugation via Primary Amines

**Figure 1:**
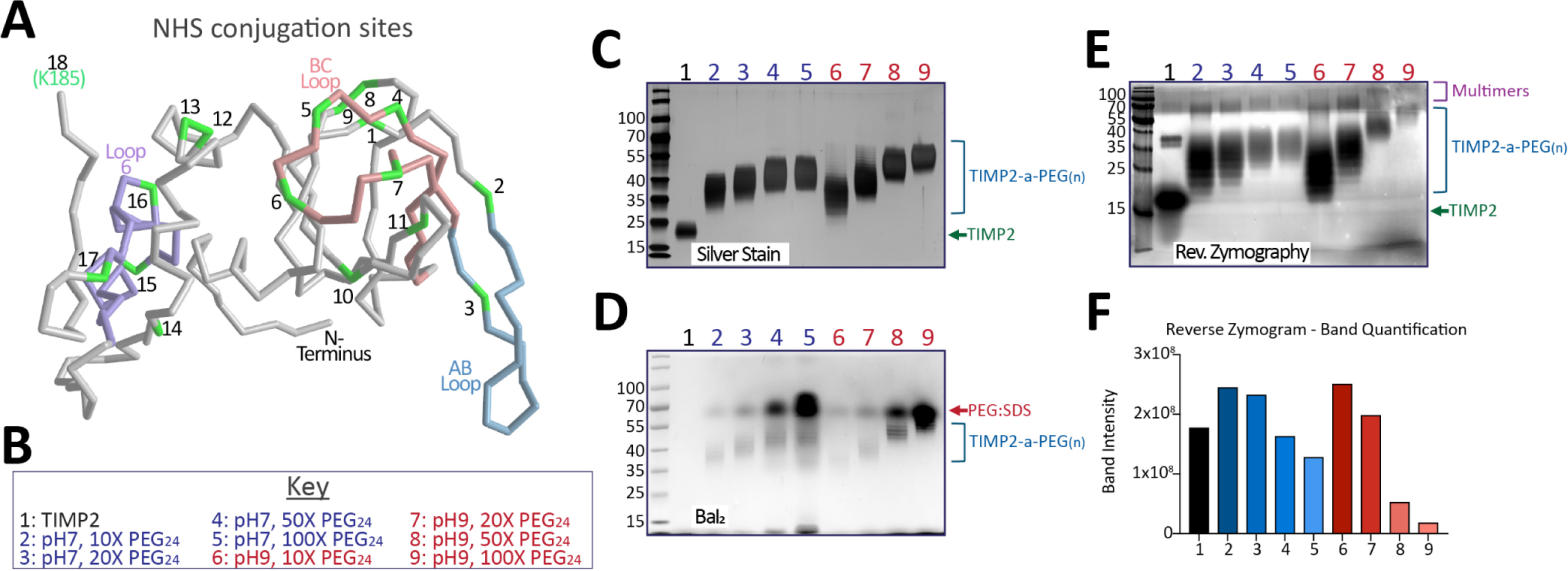
Primary amine conjugation of PEG_24_ (1 kDa) to TIMP2. (A) Crystal structure of TIMP2 (truncated at ALA182; Protein Data Bank #1BR9), with lysines highlighted in green. (B) Key to a reaction series and images highlighting the resulting (C) silver staining, (D) Barium Iodide (BaI_2_) staining, (E) reverse zymography gels, and (F) quantification of which following conjugation of PEG_24_-NHS-ester to TIMP2.

NHS-ester coupling to primary amines (ε-amino groups of lysine residues and the α-amino group of the *N*-terminus) is a highly effective method of bioconjugation. Its widespread commercial success has led to a broad availability of reagents and extensive literature on optimal reaction conditions. TIMP2 has 19 primary amine sites (18 lysines), many of which reside in structural features that mediate key functional attributes, such as the *N*-terminus and the AB loop (MMP inhibition and affinity), the BC loop (integrin affinity), and loop 6 (IGF1R affinity) (Figure 1A, Protein Data Bank #1BR9).^29^ Due to its efficiency and the number of primary amine sites in proteins, amine PEGylation is generally performed with a molar equivalent of NHS-ester PEG. Using a commercially available ∼1.2 kDa methyl-PEG_(24)_-NHS-ester (ThermoFisher, #22687), we assessed the efficiency of labeling across different reaction conditions (Figure 1B–D). In alkaline conditions (pH 9), it is likely that more primary amines are deprotonated, increasing reaction efficiency by making the amino groups more nucleophilic and pushing the labeling reaction to completion. This can be appreciated in the silver-stained SDS-PAGE gel and molecular weight comparison, with pH 9 conditions supporting the highest ratio of PEG:TIMP2 conjugations (Figure 1Cand Table 1). Barium iodide (BaI_2_) is an effective method for staining PEG groups and is also used to highlight the range of isomers of the resulting TIMP2-amine-PEG population (TIMP2-a-PEG(_*n*_)). This method also exposes residual PEG in these scouting reactions, which presents as a high molecular weight band in SDS-PAGE BaI_2_ staining due to SDS-PEG interactions^30^ (Figure 1D). Furthermore, we show that functional retention (MMP inhibition) is directly related to the level of PEGylation, observed through semiquantitative reverse zymography, revealing that higher levels of PEGylation translate to a reduction in MMP inhibition (Figure 1E,F). Similar effects were observed when amine PEGylating TIMP2 with PEG_4_ (0.33 kDa), providing further evidence that high levels of amine modification through PEGylation translate to a decrease in MMP inhibitory capabilities (Figure S1). Multimers are also apparent in these reverse zymograms, which is a common feature of recombinant TIMP proteins in reverse zymography.^31^

**Table 1:**
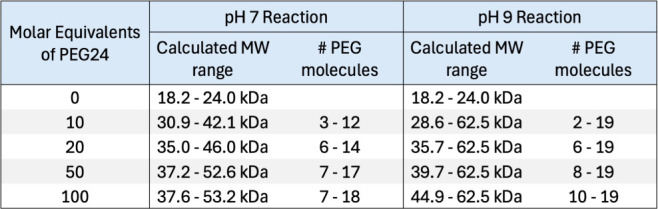
Optimization of Primary Amine Conjugation of PEG_24_ (1 kDa) to TIMP2

**Figure 2:**
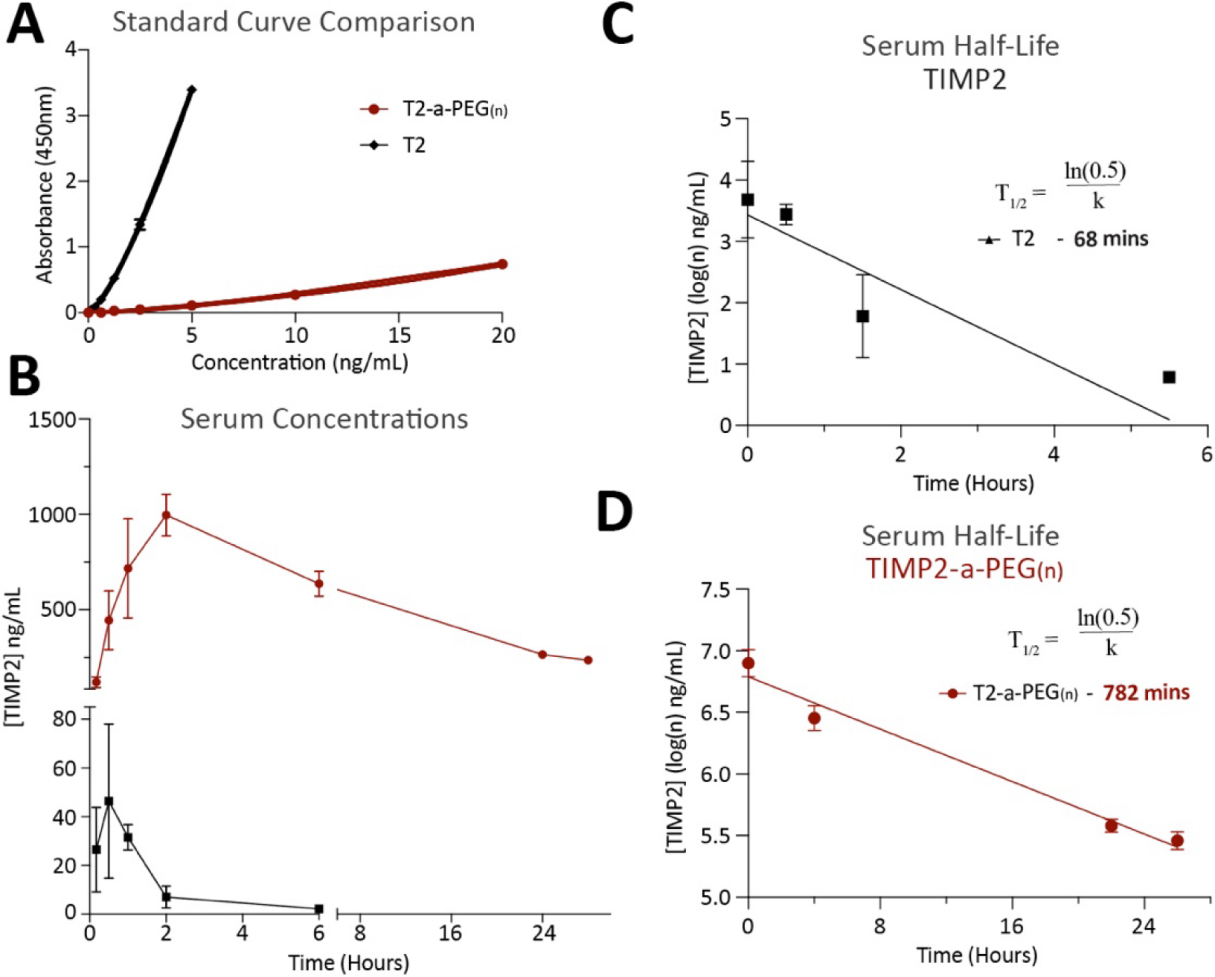
Amine PEGylated (1 kDa) TIMP2 displays an increased serum half-life. (A) Standard curve comparison between TIMP2 and amine PEGylated TIMP2 (TIMP2-a-PEG(_*n*_)). (B) Comparison of calculated serum concentrations of TIMP2 and TIMP2-a-PEG(_*n*_) between 0 and 28 h. (C,D) Serum half-life of (C) TIMP2 and (D) TIMP2-a-PEG(_*n*_), calculated after peak concentrations at 30 min (TIMP2) and 2 h (TIMP2-a-PEG(_*n*_)).

Based on the previous experiments, we determined that a 20× excess of PEG at pH 7 would be the best option for scaling up our labeling procedure and enabling broad PEGylation with a limited effect on function. Following labeling, Zeba Spin Desalting Columns (ThermoFisher, no. A43879) were utilized to remove excess PEG and buffer exchange into PBS pH 7.4, with subsequent endotoxin testing confirming that the formulation was endotoxinfree. To assess the consequences of amine PEGylation on TIMP2 circulating half-life, we replicated previous methods utilized in the lab for the delivery of therapeutic TIMP2 by intraperitoneal injection of 0.2 μg/g with a 300–400 μL injection volume (depending on mouse weight).^4,7^ Tail vein blood collections were taken at minute 0 (preinjection), 10, 30, 60, 120, 360 (6 h), 1440 (24 h), and 1680 (28 h) for analysis by ELISA. Based on our understanding of TIMP2 clearance, which is mostly renal (Figure S2),^32^ and the biomechanics of glomerular filtration that has a molecular weight threshold somewhere between 30 and 50 kDa,^33,34^ our analysis assumes that the elimination phase begins immediately after peak plasma concentration. Initial observations reveal that TIMP2-a-PEG(_*n*_) displays a significant reduction in immunoreactivity. This is highlighted in the comparison between standard curves for TIMP2-a-PEG(_*n*_) and TIMP2, revealing a significant decrease in ELISA sensitivity when TIMP2-a-PEG(_*n*_) was assayed (Figure 2A). Despite reduced detection sensitivity, TIMP2-a-PEG(_*n*_) exhibits a significantly extended serum half-life with strong detection observed even at the final time point of 28 h and a calculated half-life of 782 min (*n* = 4 mice per group) (Figure 2B,C,D). In comparison, TIMP2 alone has a half-life of 68 min, representing an 11.5-fold increase in circulating half-life after amine PEGylation. Profoundly, the peak TIMP2-a-PEG(_*n*_) abundance is 10–20 times the maximum detected of unconjugated TIMP2, with levels detected at 28 h still being around 5 times the maximum of unconjugated TIMP2. Furthermore, TIMP2-a-PEG(_*n*_) exhibits a delayed *T*max, which we suggest is associated with slower absorption of PEGylated TIMP2 into circulation after intraperitoneal injection (Figure 2B).

### 10 kDa PEG Conjugation via Histidines

**Figure 3:**
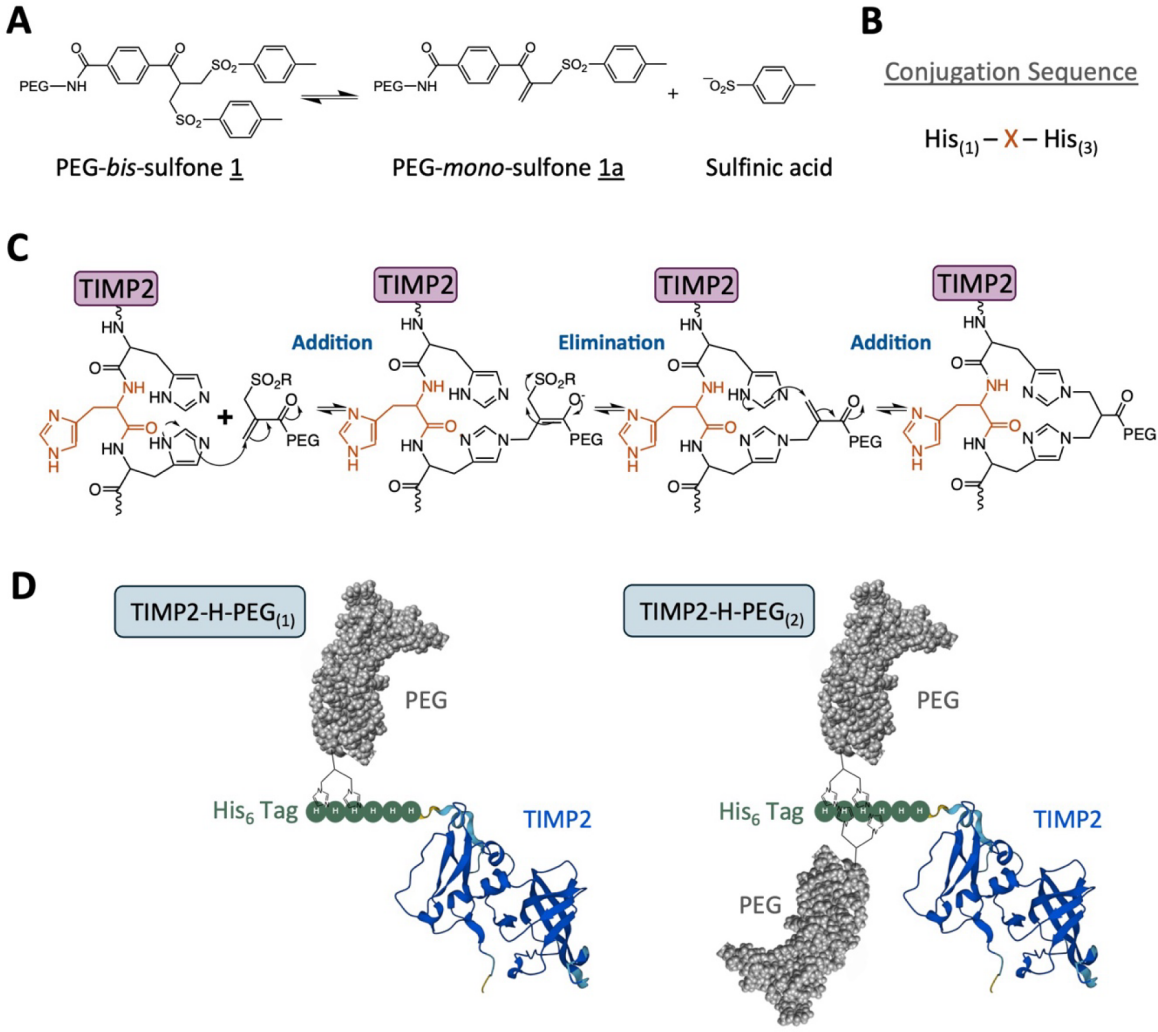
*Bis***-**sulfone reaction scheme for chemical conjugation to histidines. (A) *Bis*-sulfones undergo elimination of a sulfinic acid to give monosulfone. (B) Target conjugation sequence for *bis*-sulfone linkage to histidines. (C) Proposed reaction steps involved in *bis*-sulfone conjugation to target histidines. (D) Proposed predominant products of PEG-*bis*-sulfone conjugation to TIMP2.

**Figure 4:**
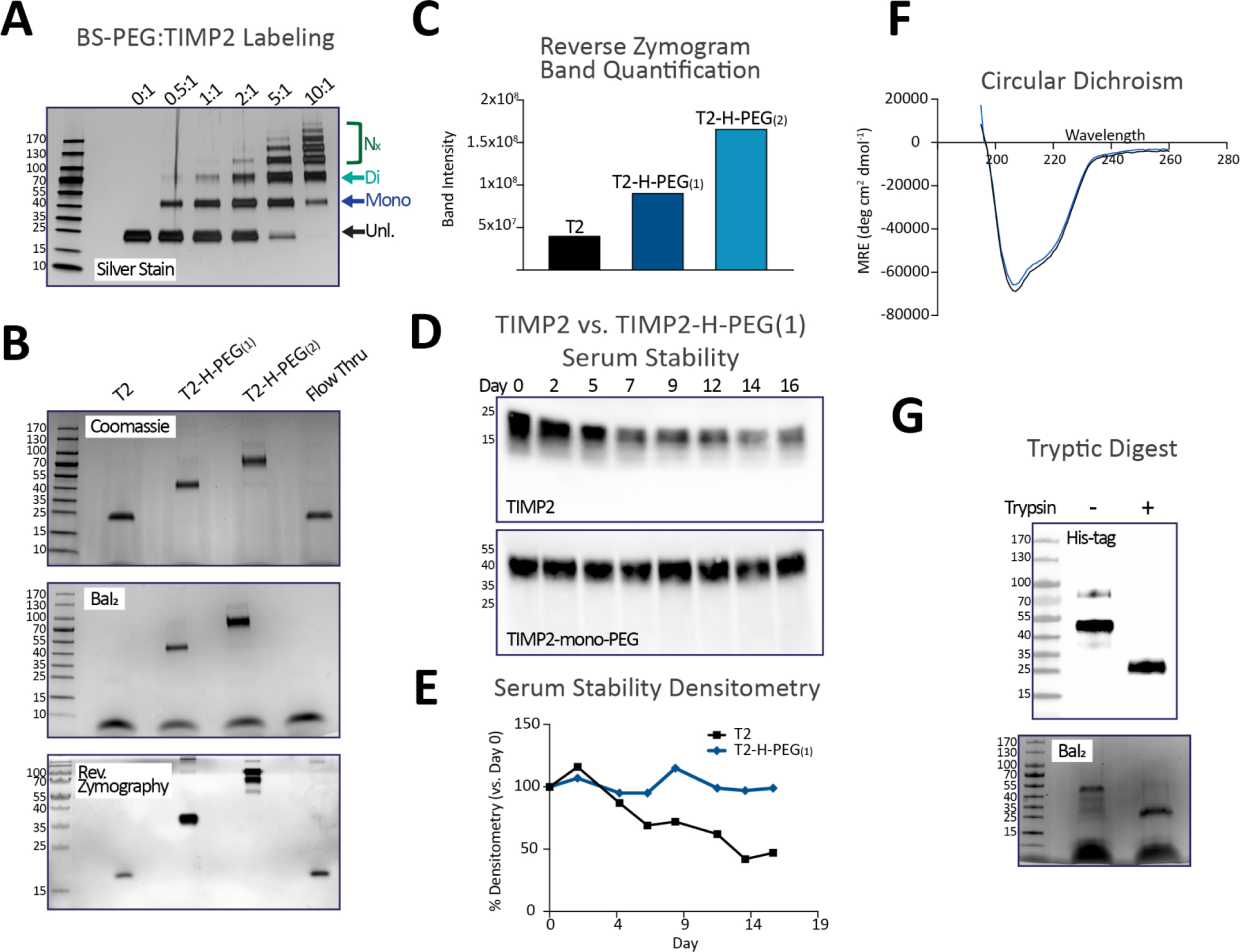
Histidine labeling of TIMP2 using *bis*-sulfone PEG (10 kDa). (A) Reaction series with varying ratios of TIMP2-to-BS-PEG. Unlabeled (Unl.), TIMP2-H-PEG_(1)_, TIMP2-H-PEG_(2)_, and excess (*N*_*x*_) PEGylated versions are highlighted. (B) Analysis of purified BS-PEG labeled TIMP2 by Coomassie stain, Barium Iodide (BaI_2_) stain, and reverse zymography. (C) Quantification of the reverse zymogram band intensity. (D) Immunoblot and (E) densitometry illustrating the stability of TIMP2 versus TIMP2-H-PEG_(1)_ in human serum at 37 °C. (F) Circular dichroism of TIMP2 versus TIMP2-H-PEG_(1)_. (G) Tryptic digestion of TIMP2-H-PEG_(1)_. followed by immunoblot using an anti-6×-His antibody and Barium Iodide (BaI_2_) staining, to demonstrate that the PEG is present on the his-tag.

*Bis*-sulfone conjugation to histidines relies on the absence of free thiol groups and the presence of a polyhistidine sequence (His-*x*-His, where *x* is any amino acid), with the *bis*-sulfone first undergoing an elimination of a sulfinic acid to give monosulfone, which then mediates conjugation to proximal histidines through *bis*-alkylation (Figure 3A,B,C). The TIMP2 utilized in these studies contains a six-histidine tag, allowing a maximum of two PEG conjugates per His_6_ tag (Figure 3D). In solution, conjugation of a single 10 kDa PEG yields an approximately 20 kDa increase in observed molecular weight through SDS-PAGE due to PEG molecule hydration. To determine the optimal conditions for producing mono- and di-PEGylated TIMP2 (TIMP2-H-PEG_(1)_ and TIMP2-H-PEG_(2)_), we performed a scouting reaction with different ratios of PEG-*bis*-sulfone:TIMP2 (Figure 4A). As expected, increasing the amount of PEG-*bis*-sulfone increased the level of PEG conjugation to TIMP2; however, this also increased the abundance of off-target PEGylation that likely occurs at other, less favored nucleophilic residues. To favor the purification of TIMP2-H-PEG_(1)_ and TIMP2-H-PEG_(2)_, we utilized a 3:1 ratio of PEG-*bis*-sulfone:TIMP2 in a scaled-up reaction. Following purification, we achieved 14% and 4% yields of TIMP2-H-PEG_(1)_ and TIMP2-H-PEG_(2)_, respectively, highlighting the more limited efficiency of site-specific PEGylation. Nonetheless, unconjugated TIMP2 can be retained (“Flow Thru”) and is functional, as determined by reverse zymography (Figure 4B). Furthermore, the final TIMP2-H-PEG_(1)_ and TIMP2-H-PEG_(2)_ show good purity and retention of function, with the latter visualized through reverse zymography (Figure 4B,C). Considering the achieved yields, we focused further studies on TIMP2-H-PEG_(1)_. Assessment of the stability of TIMP2-H-PEG_(1)_ in human serum over 16 days at 37 °C highlights that PEGylation significantly increases the stability of the protein, as visualized through immunoblotting and background-normalized densitometry of the resulting image (Figure 4D,E). We utilized spectrophotometry (absorbance at 280 nm) to quantify both TIMP2 and TIMP2-H-PEG_(1)_, with equal amounts being used for analysis by circular dichroism (CD). To correct for potential errors in protein concentration determination, UV absorbance data in the range 205–225 nm were used to adjust the relative CD signals for comparative analysis. The results indicated that the CD spectra of TIMP2 and TIMP2-H-PEG_(1)_ were highly similar, with nearly identical spectral shapes. This finding suggests that PEGylation and mild reducing conditions do not significantly alter the structural integrity of TIMP2 (Figure 4F). Finally, we show that PEGylation is largely specific to the His_6_ tag by performing an overnight tryptic digest of TIMP2-H-PEG_(1)_ followed by immunoblotting for the His_6_ tag and BaI_2_ staining. Full cleavage of the protein would produce a 15 amino acid product after K185 that encompasses the His_6_ tag. This analysis revealed that the His_6_ tag remains conjugated to PEG after digestion, running at approximately 25 kDa in SDS-PAGE (Figure 4G).

**Figure 5:**
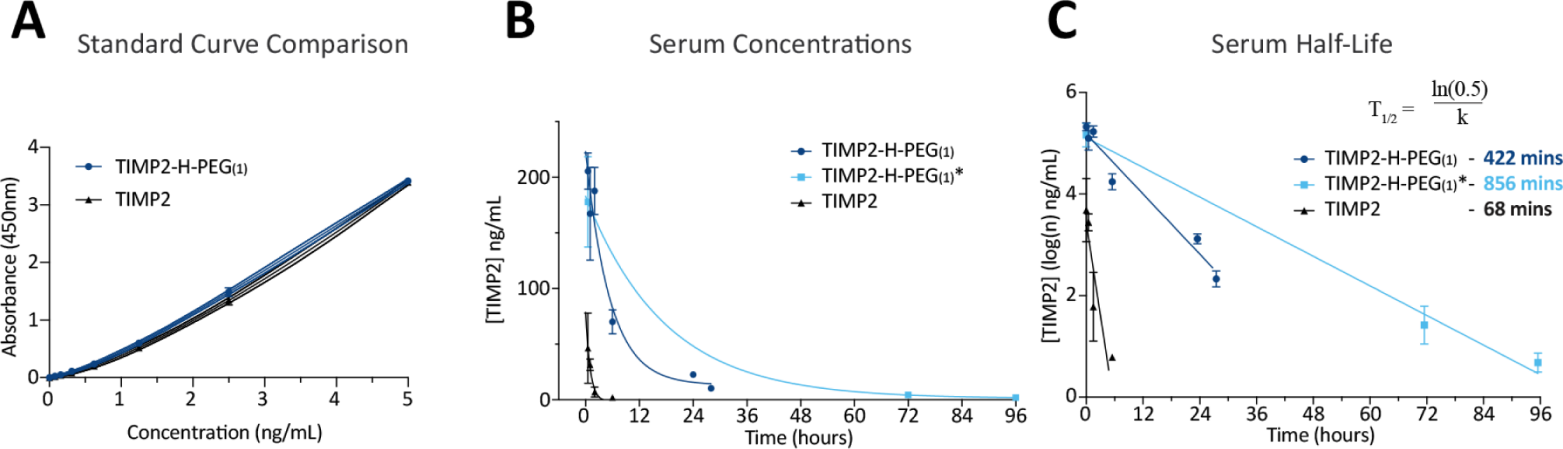
TIMP2-H-PEG_(1)_ displays an increased serum half-life. (A) Standard curve comparison between TIMP2 and TIMP2-H-PEG_(1)_. (B) Comparison of calculated serum concentrations of TIMP2 and TIMP2-H-PEG_(1)_ between 0 and 28 h, and 0 and 96 h (TIMP2-H-PEG_(1)_*). (C) Serum half-life of TIMP2, TIMP2-H-PEG_(1)_ (0–28 h), and TIMP2-H-PEG_(1)_* (0–96 h), calculated after peak concentrations at 30 min.

Endotoxin testing confirmed that TIMP2-H-PEG_(1)_ was endotoxinfree (<0.03 EU/mL), so we reproduced the previous animal studies to determine the circulating half-life of TIMP2-H-PEG_(1)_. The animal experiment was performed in duplicate (*n* = 4), with the second experiment (TIMP2-H-PEG_(1)_*) extending beyond 24 h (up to 5 days), while limiting individual animal blood draws. Importantly, we note that TIMP2-H-PEG_(1)_ does not display reduced immunoreactivity in the utilized ELISA (Figure 5A). We determined that mono-PEGylation of TIMP2 significantly increases serum half-life, calculated as 422 min (TIMP2-H-PEG_(1)_) and 856 min (TIMP2-H-PEG_(1)_*) (Figure 5B,C). Using the more accurate shorter time series, mono-PEGylation results in a 6.2-fold increase in the circulating half-life for TIMP2. TIMP2-H-PEG_(1)_ remained detectable at 4 days postinjection, with 3/4 mice presenting with 1.6–2.4 ng/mL TIMP2-H-PEG_(1)_. The peak abundance of TIMP2-H-PEG_(1)_ was 3–4 times the maximum of unconjugated TIMP2, indicating that mono-PEGylation with a 10 kDa PEG is less effective at improving PK than amine-directed PEGylation with a 1 kDa PEG.

## Conclusion

PEGylated proteins and small molecules represent a multibillion-dollar arm of the pharmaceutical industry, with numerous PEGylated products clinically approved regardless of challenges associated with safety and biological activity.^22^ Despite the convenience and efficiency of amine-targeted PEGylation, serious disadvantages remain (Table 2). Furthermore, heterogeneous mixtures of PEGylated proteins with many positional isomers are undesirable for quality control and clinical approval. Notwithstanding, most current FDA-approved PEGylated biologics utilize amine conjugation.^22^ We have previously described the therapeutic utility of TIMP2 with a C-terminal His_6_ tag,^4,7^ but its promise as a therapeutic is severely restricted by a short serum half-life, commanding daily administration to exert its beneficial effects. Histidine conjugation, though *bis*-alkylation is rarely utilized, has several key benefits. The conjugation target sequence of (His-*x*-His) is uncommon, and site selection is easily performed with standard mutagenesis. The His_6_ tag supports PEG conjugation at four potential positions for a maximum of two PEG conjugates per tag. Furthermore, conjugation is not dependent on the use of a His_6_ tag, although these have well-appreciated benefits in the early stages of protein purification. Histidine-favored conjugation has been previously reported for succinimidyl carbonate functional groups in slightly acidic conditions, although *N*-hydroxysuccinimide (NHS) esters are now more commonly utilized due to their stronger selectivity for primary amines.^35^

**Table 2:**
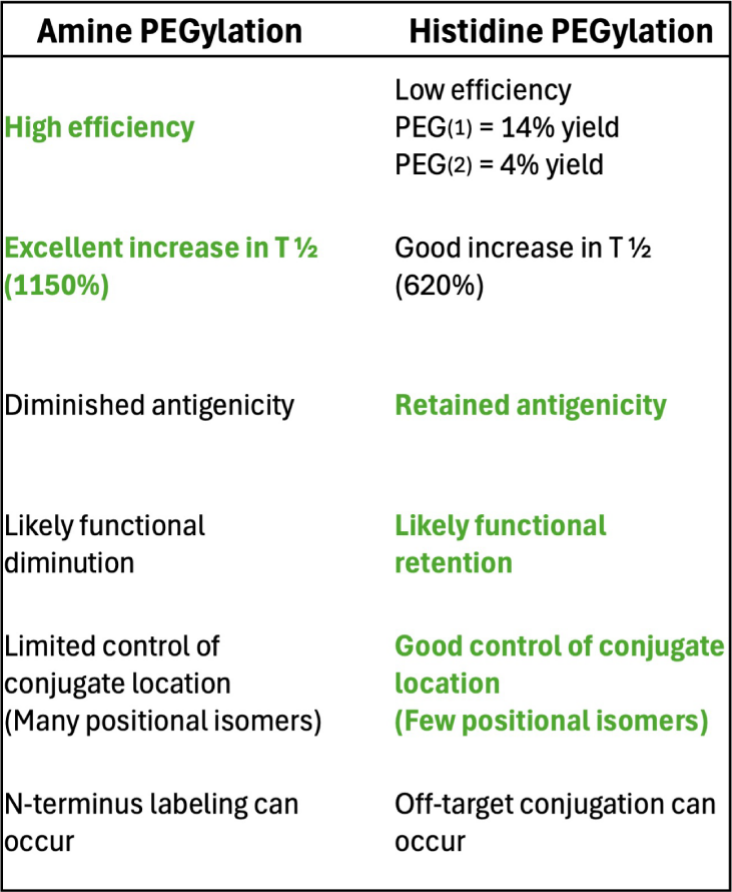
Pros and Cons of Amine PEGylation Versus Histidine PEGylation

Our findings reveal that multiple conjugations with 1 kDa PEG enable an 11.5-fold increase in the serum half-life of TIMP2, whereas a single conjugation with 10 kDa PEG improves serum half-life by 6.2-fold. These observations challenge the idea that the size of the PEG polymer, which is directly linked to hydrodynamic size, is the principal indicator of enhanced PK.^24^ Our TIMP2-a-PEG(_*n*_) presents between 6 and 14 PEG moieties per protein (Figure 1Cand Table 1), enabling a molecular weight increase similar to that of TIMP2-H-PEG_(1)_. Whether the enhanced coverage with smaller PEGs has a similar effect on hydrodynamic size compared to mono-PEGylation with a larger polymer remains to be seen. It is possible that multiple conjugations with 1 kDa PEG support a brush conformation, where adjacent PEG polymers interact with each other, promoting an elongated chain.^36^ On the contrary, with mono-PEGylation, PEG polymers are likely to exhibit a mushroom conformation due to their natural tendency to self-coil, consequently limiting the increase in hydrodynamic size.^36^ However, considering the drawbacks of amine PEGylated proteins, the number of primary amines in key domains of TIMP2, and the significant loss in immunoreactivity of TIMP2-a-PEG(_*n*_), we determined this modification to be incompatible with continued preclinical development. Site-specific PEGylation of TIMP2 produced a less profound but still impressive 6.2-fold increase in serum half-life, with TIMP2-H-PEG_(1)_ detectable 4 days postinjection. By comparison, unconjugated TIMP2 was undetectable in 3 out of 4 mice at 6 h postinjection. We also show that TIMP2-H-PEG_(1)_ retains structural and functional characteristics despite exposure to the reducing agent sodium triacetoxyhydroborate (STAB). In this context, it would be interesting to determine whether a maleimide–thiol conjugation of a free C-terminal cysteine in TIMP proteins would prove to be successful and efficient following STAB treatment. For further studies, it is feasible to pool mono- and di-PEGylated versions to increase yield and potentially improve PK. Furthermore, characterization of the off-target PEGylation sites may reveal functional retention that is compatible with preclinical development, further increasing yields. Low yields pose a significant challenge for the clinical application of histidine PEGylation. Nonetheless, unconjugated TIMP2 can be retained in the flow through. Reverse zymography illustrates that the remaining TIMP2 is functional, suggesting that this is reusable for additional conjugation reactions. It is likely that TIMP2-H-PEG_(2)_ would display a further enhanced half-life, in part through favorable conditions for a brush conformation of adjacent polymers. This was previously described with a single-domain antibody PEGylated using the same method, but with a 20 kDa PEG-*bis*-sulfone.^26^ Furthermore, it is plausible that incorporation of a His-*x*-His sequence within an exposed *N*-terminal domain structure, such as the AB-loop, may produce an optimal PK profile of therapeutic TIMP2 while retaining the benefits of site-specific histidine PEGylation.

There are limited reports describing the utilization of histidine PEGylation in other proteins, with past targets including interferon α-2a and domain antibody.^26^ TIMP2 represents a favorable target for histidine PEGylation due to its accessible C-terminal tail. It remains to be seen whether histidine PEGylation can be broadly applied to therapeutic proteins. Although initially heralded as nonimmunogenic, it has been shown that humans with limited PEG pre-exposure can develop anti-PEG immunity.^37^ Additionally, hypersensitivity reactions have been reported in multiple instances as a result of immune responses to PEGylated drugs.^23^ Histidine tags are rare in clinically approved biologic formulations due to fears of immunogenicity; however, it is feasible that PEGylation at the tag will limit immune recognition of His_6_. These potential clinical limitations are an ancillary concern with significantly improved PK and retention of bioactivity, suggesting that site-specific histidine PEGylation is a logical route forward in the preclinical development of TIMP2-based biological therapies.

## Experimental Procedures

### Amine Conjugation of PEG (1 kDa) to TIMP2

Methyl-PEG-*N*-Hydroxysuccinimide (NHS) esters of 24 repeats (1.21 kDa) (ThermoFisher, #22687) were dissolved in anhydrous DMSO at 250 mM. For scouting experiments, human TIMP2 was buffer exchanged into Hank’s balanced salt solution adjusted to pH 7 or pH 9. NHS-PEG was incubated with TIMP2 with 10, 20, 50, and 100 molar equivalents of PEG. In each case, approximately 8 μg of TIMP2 (0.75 mg/mL) was combined with the corresponding molar equivalents of PEG, gently mixed, and incubated at ambient temperature for 30 min. Samples were then subjected to a silver stain and reverse zymography analysis. Endotoxin testing was performed using a Rapid Gel Clot Endotoxin assay (ThermoFisher #A43879) to confirm endotoxin levels were below 0.03 EU/mL.

### Histidine Conjugation of PEG to TIMP2

PEG-*bis*-sulfone (10 kDa) was prepared as previously described.^38^ Scouting reactions were performed to determine the optimal pH for producing mono- and di-PEGylated TIMP2. TIMP2 and PEG-*bis*-sulfone were both resuspended in conjugation buffer (50 mM NaH_2_PO_4_, 150 mM NaCl, and 10 mM EDTA, pH 7.8). For small-scale scouting reactions, PEG:TIMP2 molar ratios of 0, 0.5, 1, 2, 5, and 10 were used to PEGylate approximately 11.7 μg of protein. The appropriate amount of PEG and conjugation buffer was combined with TIMP2 in each case to achieve a final protein concentration of 0.2 mg/mL. The samples were gently mixed and then incubated at ambient temperature for 16 h. The samples were then cooled to 4°C, and sodium triacetoxyborohydride (STAB) was added to a final concentration of 25 mM. The samples were gently mixed and allowed to rest for 40 min. These samples were then subjected to silver stain and reverse zymography analysis.

A PEG-*bis*-sulfone molar equivalent of 3:1 was chosen for scaled-up production of PEGylated TIMP2. Approximately 1.36 mg of TIMP2 in conjugation buffer was combined with the appropriate amount of PEG to achieve a final protein concentration of 0.6 mg/mL. The sample was vortexed and treated with STAB as described for the small-scale reaction.

### Purification of TIMP2-H-PEG_(1)_ and TIMP2-H-PEG_(2)_

Approximately 2 mL of the PEGylated sample was buffer exchanged using a 15 mL 10 K MWCO spin concentrator (Amicon). The ∼2 mL sample was placed into the concentrator, and the remaining ∼15 mL volume was topped up with 100 mM sodium acetate trihydrate, pH 4, and centrifuged at 3234 *g*. This was repeated twice for a total of ∼45 mL of buffer through the concentrator, resulting in an ∼2 mL final sample.

The sample, Buffer A, and Buffer B inlets, sample loop, and fraction collector of a Bio-Rad NGC were rinsed and incubated in 0.5 M NaOH for 30 min to remove endotoxins from the system. The lines were then rinsed with clean, filtered water, followed by 100 mM sodium acetate trihydrate, pH 4 (Buffer A). A 5 mL HiTrap MacroCap SP (Cytiva) was rinsed with 30 column volumes (CVs) of water and then equilibrated with 10 CVs of Buffer A.

The sample was loaded into a syringe and injected onto a clean 2 mL sample loop. Purification was performed with the method as follows: the sample was loaded from the loop onto the column with 3.5 mL of Buffer A, collected in bulk via the fraction collector, the column was washed with Buffer A for 1 CV, collected in bulk, and the bound protein was eluted with a 20 CV gradient from 0 to 100% 100 mM sodium acetate trihydrate, pH 4 + 1 M NaCl (Buffer B), collected 0.5 mL fractions in a 1.2 mL 96-well plate, followed by 1 CV at 100% B, and a 1 CV 100–0% B gradient. Fractions from the purification were analyzed via a 4–20% SDS-PAGE Coomassie-stained gel (Bio-Rad). The fractions containing the desired PEGylated species were pooled and buffer exchanged as before into Buffer A using ∼10 times the volume of buffer to the sample. The concentration of the final pools was determined using a NanoDrop One spectrophotometer (ThermoFisher), with a molecular weight (22.58 kDa) and extinction coefficient (33180). A LAL endotoxin test (Charles River) was performed on a representative sample to confirm the lack of detectable endotoxin.

### Reverse Zymography

Assessment of the MMP inhibitory function was assessed by reverse zymography. This was performed by the generation of 15% polyacrylamide gels with 8 mg of gelatin and 5 μg/1 μg of MMP2 or MMP9 embedded, respectively. Nonreduced samples were loaded in 2× or 4× Laemmli buffer and run for 90–120 min at 150 V (until the dye reaches the bottom). Zymograms were removed and incubated in 100 mL of 2.5% Triton ×-100 for 1 h, replacing the solution 3 times. The buffer was then decanted and replaced with 100 mL of enzyme buffer (50 mM Tris-HCl, pH 7.5, 200 mM NaCl, 5 mM CaCl_2_, 0.02% Brij-35), then incubated for 20 h at ambient temperature. After incubation, the zymogram was incubated with 0.5% Coomassie blue G-250 in 30% MeOH, 10% acetic acid solution for 2–4 h followed by multiple changes in destain solution (30% MeOH, 10% acetic acid) to visualize bands. Coomassie blue stained bands are indicative of MMP inhibition at the observed molecular weight. Image analysis was performed by using Image Lab (Bio-Rad).

### Barium Iodide Staining

Barium iodide staining visualizes PEG within polyacrylamide gels.^39^ Approximately 1 μg of each PEGylated species is loaded into a polyacrylamide gel and separated via SDS-PAGE. The gel is immediately placed into a 5% (w/v) BaCl_2_ solution with gentle rocking for 10 min. Next, 500 μL drops of a 100 mM iodine solution (100 mM resublimed I_2_, 300 mM KI) are added with gentle shaking by hand until bands appear clearly but before overstaining by visual inspection. The gel is then rinsed for 20–40 s in ultrapure H_2_O to remove excess stain and imaged immediately. Afterward, the gel can be rinsed repeatedly with ultrapure H_2_O to remove the iodine stain completely for subsequent Coomassie blue staining overnight.

### Circular Dichroism

Circular dichroism (CD) spectra were recorded using a Chirascan Q100 spectrometer (Applied Photophysics, U.K.) with 1 mm path length cuvettes. Data were collected at 1 nm wavelength intervals with an integration time of 1 s per step. Each spectrum is the average of three acquisitions, with the buffer background subtracted from the final data. The protein concentration was 8 μM in PBS, and the CD signal was normalized to the mean residue ellipticity. For accurate spectral comparison, CD signals were corrected for UV absorbance in the range of 205–235 nm to account for potential errors in concentration determination.

### Serum Stability Assay

Serum from three healthy human donors was combined in equal parts to make pooled human serum. TIMP2 or TIMP2-H-PEG_(1)_ was diluted approximately 1:50 in serum to a final concentration of 2 ng/μL. Serum dilutions were divided into aliquots of 20 μL in eight 200 μL tubes to reduce evaporative loss and placed into 37 °C. At 2–3-day intervals, a tube of each solution was removed, a sample was withdrawn, and snap frozen in liquid nitrogen for storage at −80 °C for later analysis via immunoblotting using a TIMP2 antibody (Cell Signaling Technology, #5738). Densitometric analysis was performed using ImageJ.

### Pharmacokinetic Testing

Four C57BL/6J mice (two male, two female) between 3 and 6 months of age were injected intraperitoneally with 0.2 μg/g TIMP2, TIMP2-a-PEG_(*n*)_, or TIMP2-H-PEG_(1)_, consistent with previously described therapeutic doses of TIMP2.^7^ Blood (10–20 μL) was withdrawn from the tail vein at the described intervals. Blood was diluted 1:1 with 0.2% w/v EDTA, 2% protease inhibitor cocktail (Millipore Sigma, no. P8340) in Hank’s balanced salt solution immediately after withdrawal. Blood samples were centrifuged at 1500 *g* for 15 min, and approximately 12 μL of diluted plasma was recovered, snap-frozen, and stored at −80 °C for later analysis.

The Human TIMP2 ELISA kits (Abcam, no. ab270213) were first tested to confirm immunoreactivity toward each TIMP2 isomer and no immunoreactivity toward murine TIMP2. Plasma samples were assayed at a final dilution of 1/40. Half-life (*T*1/2) is calculated as ln(0.5) divided by the slope (or elimination rate constant) *k*, the latter determined with natural log (ln) transformed concentration values followed by linear regression (*T*1/2= ln(0.5)/*k*). GraphPad Prism (version 10.4.0) was used for pharmacokinetic analysis. All animal procedures reported in this study were performed by the NCI staff. All staff and protocols were approved by the NCI Animal Care and Use Committee (ACUC, ASP No. LP-003–4) and followed federal regulatory requirements and standards. All components of the intramural NIH ACU program are accredited by AAALAC International. Procedures were approved.

### Tryptic Digest

In two tubes, 2 μg of TIMP2-H-PEG_(1)_ was reduced with 5 mM dithiothreitol at 37 °C for 30 min and then alkylated with 15 mM iodoacetamide in the dark at ambient temperature for 30 min. To one tube, 50 ng trypsin/Lys-C mix (Promega, #V5071) was added, and vehicle control was added to the other. Each tube was incubated for 16 h at 37 °C with gentle shaking. Samples were mixed with 4× Laemmli buffer, and 50 ng/1 μg were loaded into a 4–20% SDS-PAGE gel for His-tag immunoblotting and barium iodide staining, respectively.

### Urine Collection and Tissue Harvest

A single C57BL/6J mouse was injected with 0.2 μg/g TIMP2 and placed into a cage without bedding for 2 h. Fresh urine was collected over 2 h, pooled, and stored at −80 °C. The mouse was euthanized after 2 h, and kidneys were collected. Tissue was lysed using RIPA buffer plus 1% protease inhibitor cocktail (Sigma-Aldrich, #P8340) and homogenized using a GentleMACS dissociator and MACS M tubes (Miltenyi Biotech). Homogenized tissue was centrifuged at 10,000 *g* for 10 min, and the supernatant was stored at −80 °C.

## Supporting Information Available

Additional experimental data describing conjugation of PEG_4_-NHS-ester to TIMP2 (Figure S1) and detection of TIMP2 in the kidney and urine (Figure S2) (PDF)
