# Supplementary material for: Histidine Tag-Specific PEGylation Improves the Circulating Half-Life of TIMP2
Source: ACS Appl Bio Mater. 2025 Feb 21;8(3):1946–55. doi: 10.1021/acsabm.4c01385 (PMC11921907; doi:10.1021/acsabm.4c01385)
Supplement: Supplementary file 1 — mt4c01385_si_001.pdf [file mt4c01385_si_001.pdf]

Supporting Information

Histidine tag-specific PEGylation improves the circulating half-life of TIMP2

Jack Toor[1], Wiktoria R. Grabowska[2], Adam L. Johnson[3], Jane Jones[3], William G. Stetler-Stevenson[1], Hanieh Khalili[2,4], David Peeney[1*]

[1]Laboratory of Pathology, Center for Cancer Research, National Cancer Institute, Bethesda, MD, 20892, USA

[2]School of Medicine and Biosciences, University of West London, W5 5RF, UK

[3] Protein Expression Laboratory, FNLCR, NIH, Frederick, MD, 21702, USA

[4]School of Pharmacy, University College London, WC1N 1AX, UK

* Corresponding author: david.peeney@nih.gov

Key words: TIMP2, PEGylation, Pharmacokinetics, Biologics, Therapeutics.

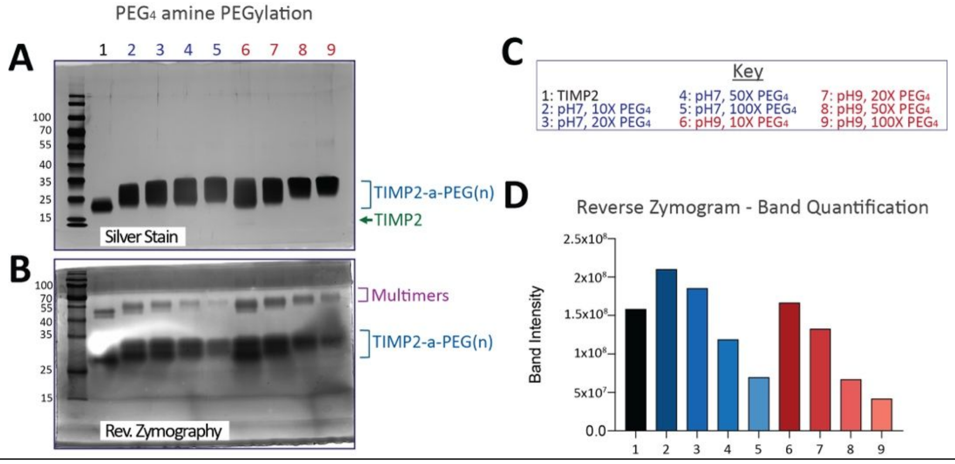

Figure S1. Primary amine conjugation of PEG_4 (0.33 kDa) to TIMP2. (A/B) Images highlighting the resulting Silver Stain (A) and reverse zymography (B) gels following conjugation of PEG_4-NHS-ester to TIMP2. (C) Key to the reaction series. (D) Quantification of reverse zymography bands following PEG_4-NHS-ester conjugation to TIMP2.

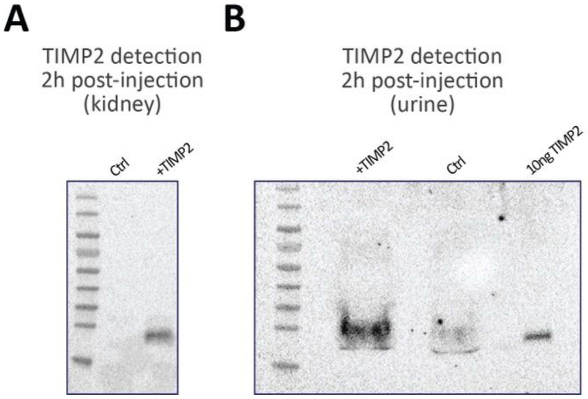

Figure S2. TIMP2 detection in the kidneys and urine 2 hours post intraperitoneal injection. Immunoblot images showing TIMP2 detection in the kidneys (A) and urine (B) of a TIMP2-treated mouse.
